# The Study of Pattern of Lipid Profile in Chronic Kidney Disease Patients on Conservative Management and Hemodialysis: A Comparative Study

**DOI:** 10.7759/cureus.21506

**Published:** 2022-01-23

**Authors:** Manpreet Saini, Amrita Vamne, Vijay Kumar, M S Chandel

**Affiliations:** 1 Biochemistry, Index Medical College, Hospital & Research Centre, Malwanchal University, Indore, IND; 2 Community Medicine, Phulo Jhano Medical College, Sido Kanhu Murmu University, Dumka, IND; 3 Academics, Index Medical College, Hospital & Research Centre, Malwanchal University, Indore, IND

**Keywords:** cholesterol, hemodialysis, dyslipidemia, lipid profile, ckd

## Abstract

Background: Chronic kidney disease (CKD) causes irreversible damage to the renal tissue resulting in decreased kidney function. It is known more for its morbidity than for its mortality as the deranged kidney functioning affects almost every organ system of the body. Dyslipidemia is one of the most common complications of chronic renal failure (CRF) reflected even in the early stages of CRF and usually parallels the deterioration in renal function. As a consequence, dyslipidemia as a risk factor in CKD progression should be explored and documented more. The aim of the study was to compare the pattern of lipid profile in CKD patients on conservative management with that of CKD patients on hemodialysis.

Materials and Methods: This is a cross-sectional observational study conducted in Lucknow, India, between January 2021 to May 2021 after considering inclusion and exclusion criteria. The lipid profile of 105 eligible patients was analyzed using an autoanalyzer. After generation of the proper template, data was entered in Microsoft Excel (Microsoft Corp., Redmond, Washington, United States) and analysis was done through SPSS for Windows, Version 16.0 (Released 2007, SPSS Inc., Chicago, United States).

Results: There was a statistically significant decrease in high-density lipoprotein (HDL) and an increase in triglycerides (TG) and very-low-density lipoprotein (VLDL) levels in CKD patients on hemodialysis when compared with CKD patients on conservative management. As far as total cholesterol and low-density lipoprotein (LDL) levels are concerned, they were also significantly increased in CKD patients on hemodialysis than CKD patients on conservative management.

Conclusions: Dyslipidemia progresses with the stage of CKD, so early monitoring of lipid profile in CKD patients may help in decreasing the progression of the disease and, hence, mortality in CKD patients.

## Introduction

Chronic Kidney disease (CKD) is a condition that causes damage to the kidney or its gradual impairment involving the loss of glomerular and tubular function that lasts longer than three months [1,2]. CKD has now emerged as a public health problem with a worldwide prevalence of around 8-16% [3,4] and >10% prevalence in the adult population of the United States [5]. The incidence of CKD in India, due to the lack of a national registry, is not well defined. However, it has been estimated that the prevalence of CKD in India may be up to 785 people per million population [6]. CKD gradually progresses towards end-stage renal disease (ESRD), generally in association with high cardiovascular morbidity and mortality. In fact, CKD patients are more likely to die of cardiovascular complications than ESRD [7].

Many recently published studies indicate that dyslipidemia in CKD patients may actively participate in the progression of cardiovascular disease (CVD) and in the deterioration of kidney function [6]. CVD is the leading cause of mortality in CKD patients and its incidence is even higher in patients on hemodialysis [8,9]. In the general population, dyslipidemia is a known risk factor for CVD but the relationship of dyslipidemia as a risk factor in CKD progression should be explored and documented more. Hence, this study intends to compare the pattern of lipid profile in CKD patients on conservative management with that in CKD patients on hemodialysis in the Lucknow population.

AIM: Aim of the study was to compare and correlate the pattern of lipid profile, including levels of total cholesterol, triglycerides (TG), high-density lipoprotein (HDL), low-density lipoprotein (LDL), and very-low-density lipoprotein (VLDL) in CKD patients on conservative management with that in the CKD patients on hemodialysis.

## Materials and methods

This is a cross-sectional, observational study done in different hospitals of Lucknow, Uttar Pradesh, India and data was collected with an in-vitro biophysiological method from January to May, 2021. The study protocol was approved by the Ethics Committee of Malwanchal University, Indore, Madhya Pradesh, India (MU/Research/EC/Ph.D/2020/04). Due to restrictions of coronavirus disease 2019 (COVID-19), it was estimated that 100-120 patients could be included in this study. Three hospitals were randomly selected from different areas of Lucknow and by consecutive sampling, a total of 105 patients irrespective of gender were included in this study from these hospitals.

Patients with metabolic disorders, history of smoking and alcohol, obesity (BMI ≥25 kg/m^2^), patients on hypolipidemic therapy, specific prescribed diet and lifestyle modifications were excluded as these factors could affect lipid profiles and alter our study findings. Verbal informed consent regarding this study was obtained from all the patients.

Stages of CKD patients were classified based on glomerular filtration rate (GFR) as per the National Kidney Foundation Kidney Disease Outcomes Quality Initiative (NKF KDOQI)™. Subjects were divided into two groups, i.e., Group A and Group B. Group A included 55 subjects with CKD of stage I to IV (GFR of 15-59 ml /min / 1.73m2) on conservative management and Group B included 50 subjects with CKD of stage V (GFR < 15ml/min / 1.73m2) on hemodialysis. Blood samples were collected in sterile tubes from patients in both groups taking all aseptic precautions for analysis of levels of serum total cholesterol, TG, HDL, LDL, and VLDL by HumaStar 200 automated analyzer (Human Diagnostics Worldwide, Wiesbaden, Germany) using an enzymatic colorimetric method.

Statistical analysis

Data collected were entered in Microsoft Excel (Microsoft Corp., Redmond, Washington, United States) after the generation of a proper template. It was then imported into SPSS for Windows, Version 16.0 (Released 2007, SPSS Inc., Chicago, United States) and analysis was done. Different parameters of lipid profile were expressed in terms of mean±SD. Chi-square test was applied to see the association of age and sex with CKD patients on conservative management and on hemodialysis and the unpaired t-test was used to see the difference in the mean values of lipid analytes. A P-value of <0.05 was considered to be statistically significant.

## Results

Table 1 shows the age and sex of the study participants. Their age varied from 28 years to 77 years with the mean age of 46.42±12.88 years. A total number of 105 subjects were included in the study and divided into two groups. Group A included 55 CKD patients on conservative management and Group B included 50 CKD patients on hemodialysis. CKD patients in both the groups were mostly in the age group of 41-60 years. The mean age of CKD patients on conservative management was 44.02±13.20 years and that of CKD patients on hemodialysis was 49.08±12.11 years. The difference in mean age between two groups was found to be statistically significant (P<0.05). In both the groups, male patients were more than females. Group A comprised 42 (76.4%) males and 13 (23.6%) females while Group B comprised 32 (64.0%) males and 18 (36.0%) females. The association of age and sex with CKD patients on conservative management and on hemodialysis was not significant.

**Table 1:** Age and sex of CKD patients on conservative management and CKD patients on hemodialysis CKD: chronic kidney disease

Variable		Group A: CKD patients on conservative management	Group B: CKD patients on hemodialysis	Chi-square value	P valve
Age	20-40	16	12	1.132	0.56
	41-60	32	28		
	>60	7	10		
Sex	Male	42	32	1.924	0.16
	Female	13	18		

**Figure 1:**
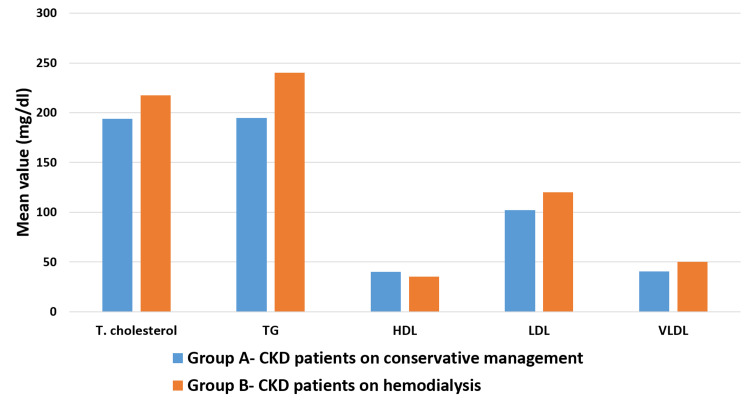
Comparison of pattern of lipid profile of CKD patients on conservative management and CKD patients on hemodialysis T. Cholesterol: total cholesterol; TG: triglycerides; HDL: high-density lipoprotein; LDL: low-density lipoprotein; VLDL: very-low-density lipoprotein.

Figure 1 shows the mean of different parameters of lipid profile of both the groups. The mean values of total cholesterol, TG, HDL, LDL, and VLDL in Group A were found to be 194.00 ± 6.12 mg/dl, 194.64 ± 21.20 mg/dl, 39.99 ± 6.47 mg/dl, 101.94 ± 21.89 mg/dl, and 40.62 ± 4.80 mg/dl, respectively, and in Group B, the mean values of total cholesterol, TG, HDL, LDL and VLDL were found to be 217.60 ± 9.47 mg/dl, 240.20 ± 35.40 mg/dl, 35.23 ± 6.05 mg/dl, 120.22 ± 38.53 mg/dl, and 50.32 ± 8.00 mg/dl, respectively.

To compare the pattern of lipid profile in both the groups, unpaired t-test was done and P-value was calculated. The P-value < 0.05 was considered significant. As can be seen in Table 2, total cholesterol and LDL values were significantly lower (P-value < 0.05) in Group A than in Group B CKD. TG and VLDL values of Group A were also lower than in Group B and this was also found to be statistically significant (P-value <0.001). On comparison, it was found that HDL was the only lipoprotein higher in Group A than in Group B and this difference was statistically significant (P-value <0.001).

**Table 2:** Comparison of pattern of lipid profile of CKD patients on conservative management (Group A) and CKD patients on hemodialysis (Group B) CKD: chronic kidney disease; TG: triglycerides; HDL: high-density lipoprotein; LDL: Low-density lipoprotein; VLDL: very-low-density lipoprotein.

Serum lipids (mg/dl)	Group A – CKD patients on conservative management (Mean ±SD)	Group B – CKD patients on hemodialysis (Mean ± SD)	P valve
Total cholesterol	194.0 ± 6.12	217.60 ± 9.47	<0.001
TG	194.64 ± 21.20	240.20 ± 35.40	<0.001
HDL	39.99 ± 6.47	35.23 ± 6.05	<0.001
LDL	101.94 ± 21.89	120.22 ± 38.53	<0.05
VLDL	40.62 ± 4.80	50.32 ± 8.00	<0.001

## Discussion

The study was conducted to compare the pattern of lipid profile in CKD patients on conservative management (Group A) with the CKD patients on hemodialysis (Group B). The mean age of CKD patients in the current study was 46.42±12.88 years, which was almost similar to study done by Adejumo et al. in which mean age was found to be 46.98±16.81 years [10]. Kumari and Srinivas also found the mean age of CKD patients to be 45.28 years in their study [11]. The proportion of males and females in the present study was 70.5% and 29.5%, respectively, which is also similar to the study by Kumari and Srinivas, where males and females constituted 68% and 32%, respectively [11]. In our study, the most common findings were low HDL levels and hypertriglyceridemia along with a modest increase in LDL, VLDL, and total cholesterol in Group B as compared to Group A, which is supported by the study of Choudhary in which hypertriglyceridemia in stage V CKD patients was noticed [12]. Zolezzi et al. also reported increased total cholesterol, triglycerides, and decreased HDL in patients on hemodialysis [13]. Morena et al., in their study, stated that hemodialysis patients are exposed to several atherogenic factors resulting from qualitative and functional lipid abnormalities, including triglyceride-rich particles, increased susceptibility to LDL oxidation and impairment of HDL protective effects [14]. However, this study is in contrast to the study by Lokesh who found low levels of total cholesterol, TG, HDL, VLDL, and LDL among patients undergoing hemodialysis when compared to controls [15].

In our study we found TGs to be high in CKD patients on hemodialysis as compared to CKD patients on conservative management, which are well documented by Singh [16] and is also strongly supported by Zolezzi et al., who reported high TG in CKD patients on hemodialysis [13]. The increase in TGs in hemodialysis patients is more compared to non-hemodialysis patients due to heparin used in hemodialysis, which inhibits lipoprotein lipase, responsible for the hydrolysis of triglycerides. The other reason is the direct inhibitory effect of uremic ‘toxins’ on enzymes of lipid metabolism. The present study observed increased VLDL in CKD patients on hemodialysis, which is in accordance with the study of Singh [16]. Similar findings were observed by Mikolasevic I [17]. The possible increase is in linearity with hypertriglyceridemia but is still a matter of dispute. The increased VLDL cholesterol concentration in CKD is because of delayed catabolism of VLDL. In this study, LDL levels in CKD patients on hemodialysis was found to be more as compared to CKD patients on conservative management, which is similar to the study of Singh [16]. Increased LDL sub-fractions in hemodialysis patients were also found in the study by Morena et al. [14]. The current study observed HDL levels to be low in CKD patients on hemodialysis, which is consistent with a study done by Ganta [18]. Our study is also strongly supported by the paper by Barbagallo et al. [19]. A possible mechanism involved in low HDL levels is that uremic patients generally exhibit decreased levels of apolipoprotein AI and AII. Decreased activity of lecithin-cholesterol acyltransferase (LCAT) and increased activity of cholesterol ester, facilitate the transfer of cholesterol esters from HDL to TG-rich lipoproteins, which reduces HDL cholesterol. In addition to this, impaired reverse cholesterol transport decreased HDL protective capacity against oxidative stress could be the reason for decreased HDL in hemodialysis. As far as total cholesterol levels are concerned, they were found to be elevated in CKD patients on hemodialysis in our study, which is in accordance with the findings by Kumari and Srinivas [11] but most of the studies did not observe hypercholesterolemia. The possible reason for increased total cholesterol levels in our study could be due to an increase in cholesterol-containing lipid fractions.

The limitation of our study was a small sample size due to restrictions of COVID-19.

## Conclusions

This study observed a decrease in HDL levels and an increase in total cholesterol, TG, LDL, and VLDL levels in CKD patients on hemodialysis as compared to CKD patients on conservative management. Alteration in lipid and carbohydrate metabolism along with the progression of stage of CKD can lead to increased risk of atherogenesis leading to poor prognosis and increased mortality rate of the disease. This emphasizes the need of periodic monitoring and maintenance of lipid profiles in CKD patients even at the earliest stage to prevent cardiovascular morbidity and mortality. Despite so many studies on lipid profile in CKD patients, the trend of lipoprotein pattern of CKD patients on hemodialysis remains inconsistent and so it is recommended that more focus should be given to multi-centric studies to have a conclusive view on pattern of lipid profile in CKD patients.
